# CD44 Staining of Cancer Stem-Like Cells Is Influenced by Down-Regulation of CD44 Variant Isoforms and Up-Regulation of the Standard CD44 Isoform in the Population of Cells That Have Undergone Epithelial-to-Mesenchymal Transition

**DOI:** 10.1371/journal.pone.0057314

**Published:** 2013-02-20

**Authors:** Adrian Biddle, Luke Gammon, Bilal Fazil, Ian C. Mackenzie

**Affiliations:** 1 Blizard Institute, Barts and the London School of Medicine and Dentistry, Queen Mary University of London, London, United Kingdom; 2 Gade Institute, University of Bergen, Haukeland University Hospital, Bergen, Norway; The University of Texas M.D Anderson Cancer Center, United States of America

## Abstract

CD44 is commonly used as a cell surface marker of cancer stem-like cells in epithelial tumours, and we have previously demonstrated the existence of two different CD44^high^ cancer stem-like cell populations in squamous cell carcinoma, one having undergone epithelial-to-mesenchymal transition and the other maintaining an epithelial phenotype. Alternative splicing of CD44 variant exons generates a great many isoforms, and it is not known which isoforms are expressed on the surface of the two different cancer stem-like cell phenotypes. Here, we demonstrate that cancer stem-like cells with an epithelial phenotype predominantly express isoforms containing the variant exons, whereas the cancer stem-like cells that have undergone an epithelial-to-mesenchymal transition down-regulate these variant isoforms and up-regulate expression of the standard CD44 isoform that contains no variant exons. In addition, we find that enzymatic treatments used to dissociate cells from tissue culture or fresh tumour specimens cause destruction of variant CD44 isoforms at the cell surface whereas expression of the standard CD44 isoform is preserved. This results in enrichment within the CD44^high^ population of cancer stem-like cells that have undergone an epithelial-to-mesenchymal transition and depletion from the CD44^high^ population of cancer stem-like cells that maintain an epithelial phenotype, and therefore greatly effects the characteristics of any cancer stem-like cell population isolated based on expression of CD44. As well as effecting the CD44^high^ population, enzymatic treatment also reduces the percentage of the total epithelial cancer cell population staining CD44-positive, with potential implications for studies that aim to use CD44-positive staining as a prognostic indicator. Analyses of the properties of cancer stem-like cells are largely dependent on the ability to accurately identify and assay these populations. It is therefore critical that consideration be given to use of multiple cancer stem-like cell markers and suitable procedures for cell isolation in order that the correct populations are assayed.

## Introduction

Cancer stem-like cells (CSCs) are defined as a subpopulation of tumour cells that have both tumour-initiating ability and the ability to reconstitute the cellular heterogeneity typical of their tumours of origin [1]. Cells with such properties have been found in cancers arising in haematopoietic, epithelial, neural and other tissues [2]–[5]. Marked clinical interest in CSCs has arisen due to reports of unique CSC properties that implicate them in local tumour invasion, metastasis, and therapeutic resistance [6]–[9]. Although initially CSCs were thought typically to represent a relatively small fraction of the total number of tumour cells, subsequent reports suggest that their number may vary quite widely both within a given type of tumour and between differing types of tumours [10], [11]. Such differences in the size of the CSC fraction within tumours is reported to be of prognostic significance [12], [13] but the accuracy of methods currently used to determine the proportion of cells with CSC properties is uncertain.

CSCs are typically identified by their higher levels of expression of certain cell surface molecules but other characteristics, such as greater expression of ABC transporters and of molecules such as Aldh1, have also been used to isolate cells with CSC characteristics [14], [15]. The tumour initiating properties of cell fractions is typically assessed by murine transplantation and this has been taken as the “gold standard” functional assay [1]. However, the fraction of cells found to initiate tumours on transplantation is influenced markedly not only by the method and site of transplantation but also by the type of recipient mouse [10]. In addition, it is unclear how the composition of test populations is influenced by the method used for their isolation, and there has been little investigation of the effects of differing methods of cell isolation on the levels of CSC markers subsequently detectable. In particular, many cell isolation protocols use the proteolytic enzymes trypsin [10], [16] and collagenase [3], [12], [15], yet it is not known whether these enzymes degrade cell surface molecules used to isolate CSCs.

For tumours of epithelial origin, including those of the head and neck, breast, prostate and colon, isolation of CSCs has frequently been based on their greater cell-surface expression of CD44 [2], [3], [17]–[19]. CD44 is a multifunctional and ubiquitously expressed glyco-protein adhesion molecule derived from a gene with 18 exons, 9 of which are expressed in the standard form. Alternative splicing of the remainder generates a great many variant (CD44v) isoforms [20]. CD44 expression potentially influences stem cell behaviour by a wide range of mechanisms [21] and interaction with hyaluronan, its principal ligand, influences several signalling pathways [20], [22] and initiates signalling functions associated with nuclear translocation [23]. The abrogation of tumour growth resulting from CD44 inhibition, either *in vivo* or *in vitro,* indicates its functional significance [24], [25] and differential patterns of expression of CD44 isoforms can similarly affect cellular properties [26].

Epithelial-to-mesenchymal transition (EMT), first described as a developmental process during which epithelial cells acquire a motile mesenchymal phenotype [27], has since been shown to play an important role in the invasion of cancers of the head and neck, breast and several other tissues [28], [29]. In addition to its roles in motility, EMT has been implicated in the development of therapeutic resistance and tumour recurrence [7], [29]–[31]. Recently, we demonstrated that the CD44^high^ CSC fraction in cell lines derived from head and neck and cutaneous squamous cell carcinoma (SCC) exists in at least two phenotypic forms that differ in their patterns of cell surface marker expression and their ability to survive in suspension [32]. Although both CSC phenotypes are characterized by high levels of expression of CD44, they are distinguished by different levels of expression of epithelial-specific-antigen (ESA). CSCs with a CD44^high^ESA^high^ expression pattern have an epithelial phenotype and are proliferative, whereas CD44^high^ESA^low^ cells are CSCs that have undergone EMT and are motile. CSCs can switch between these two phenotypes through the processes of EMT and mesenchymal-to-epithelial transition (MET). Expression of EMT markers at the invasive front of colon and oral carcinoma [33], [34], correlation of EMT marker expression with tumour aggression [28], and isolation of cells expressing EMT markers from fresh tumour samples [35], indicate the *in vivo* occurrence of EMT as a mechanism associated with the spread of tumours.

We have examined whether the two different CSC phenotypes in SCC exhibit differential CD44 isoform expression. A further point of interest was that varying proteolytic sensitivities of CD44 epitopes [36] could result in differential sensitivity of the CD44 isoforms to proteolytic enzymes used for cell dissociation and thus different cellular populations being identified as CD44^high^ depending on the cell dissociation method used. We therefore investigated how the method of cell isolation from intact tumours, or release from culture, can influence their surface retention of different CD44 isoforms and, consequently, the properties of the cell fraction detected as CD44^high^ CSCs. We show that the motile CD44^high^ESA^low^ CSCs have higher expression of the standard CD44 isoform but lower expression of CD44v isoforms. Further, the standard CD44 isoform is less sensitive to enzymatic treatment than CD44v isoforms and this can greatly influence the relative degree of CD44 staining detected on ESA^high^ versus ESA^low^ CSCs, depending on the method of cell dissociation used. Comparison of various methods of cell dissociation, both from cell lines and from fresh tumours, demonstrates that they result in different populations of cells being identified as CD44^high^.

## Materials and Methods

### Cell culture

The CA1 cell line was previously derived in our laboratory from a biopsy of oral SCC of the floor of the mouth [37]. The Met1 and Met2 cell lines were derived within our Centre from matched malignant cutaneous SCC tissues [38]. All cell lines were grown in a highly supplemented epithelial growth medium (termed FAD) with 10% FBS [39].

### FACS

For FACS analyses, cells were detached from cultures at 37°C using either 1× trypsin-EDTA (PAA, #L11-004), Accutase (PAA, #L11-007) a widely used commercial product that combines protease and collagenolytic activities, or enzyme-free cell dissociation buffer (Invitrogen, #13151-014). The length of dissociation varied between these buffers, and was determined by observing the time taken for complete dissociation of the cells from the dish. Typically, trypsin required 5–10 minutes dissociation, Accutase 10–15 minutes and Enzyme Free buffer 30 minutes. One million cells per ml were then stained with antibodies at 1∶100 dilution in PBS (PAA) for 15 minutes. The DAPI nuclear dye (Sigma) was used at 1 ug/ml to exclude dead cells. The antibodies used were as follows; PE-CD44 (clone G44–26) and PE-Integrin β4 (clone 439-9B) were from BD biosciences; APC-ESA (clone HEA-125) was from Miltenyi Biotec; PE-CD44v3 (clone 3G5) was from R&D systems; CD44v4 (clone VFF11), CD44v9 (clone FW11.24) and FITC-CD44v6 (clone VFF7) were from Bender Medsystems; CD44v5 (clone VFF8), FITC-CD44v7/8 (clone VFF17) and CD44v10 (clone VFF14) were from AbD Serotec; the FITC rabbit-anti-mouse secondary antibody was from Invitrogen. The FITC- and PE- conjugated mouse IgG isotype control antibodies were from BD. Biosciences. Populations of FACS sorted cells were collected into buffer RLT (Qiagen) for RNA extraction.

### RNA extraction, cDNA synthesis and QPCR

RNA was extracted from 50000 FACS sorted cells using the RNeasy micro kit (Qiagen). Reverse transcription into cDNA was conducted using the Superscript III first strand synthesis supermix (Invitrogen), with inclusion of –RT controls. QPCR was conducted in an ABI 7500 real-time PCR system (Applied Biosystems) using Power SYBR green mix (Applied Biosystems). GAPDH was used as a reference mRNA control. QPCR cycling conditions were: 95°C for 10 mins, [95°C for 15 seconds, 60°C for 30 seconds, 72°C for 40 seconds] (40 cycles), 95°C for 60 seconds, followed by dissociation curve analysis. Reverse transcribed Human Total Reference RNA (Stratagene) was used to generate a standard curve. The primer sequences used are shown in Appendix S1 and the QPCR Ct-plots are shown in Appendix S2.

### Extraction and staining of cells from primary tumours

For analysis of cells isolated from fresh tumours, small pieces of oral SCC that were surplus to diagnostic requirements were collected, with informed consent and without patient identifiers following protocols approved by the local NHS Research Ethics Committee., and transported to the laboratory in ice-cold FAD medium. Tissues were chopped into small pieces, approximately 1×1 mm, washed in medium, and spun down. Cell suspensions were isolated using a Seward Stomacher 80 (Biomaster), an apparatus that dissociates tissues by intermittent squeezing. Each tumour sample was aliquoted into 3 individual Stomacher bags containing either FAD medium, 1× trypsin-EDTA (PAA, #L11-004), or 2.5 mg/ml Collagenase III (Sigma, #C0255). Bags were placed into the apparatus and pulsed at low speed for a total of 45 min. with medium withdrawn from the bags and replaced with fresh medium each 15 min. Cells in the fluids collected at the 3 time periods were pooled, and spun down. As only small tissue specimens were available, the number of cells obtained was too low to permit FACS analysis. The cell pellets were therefore resuspended in 0.1 ml FCS, and prepared as cell smears on polysine coated slides (VWR). Slides were air dried and fixed in ice-cold acetone/methanol (50∶50) and co-stained with DAPI to define nuclei, with rabbit anti-human keratin antibody (AbD Serotec) to identify epithelial cells, and with antibodies as listed above against CD44s and several variant isoforms (1∶100 dilution).

For each of 5 separate tumours, 4 or more random fields containing 150–300 cells were photographed using filter sets distinguishing cell staining for DAPI, keratin, and CD44. Images captured at standardized exposures were imported into PhotoShop (Adobe) and, using the Wand tool adjusted to a level of tolerance that captured visually stained cells, secondary images of isolated stained cells were generated for each fluorochrome. The 3 images of each field were then combined, slightly out of lateral register, to identify the presence or absence of staining for keratin and CD44 associated with each DAPI stained nucleus. Counts were made of total number of cell nuclei, of cells unstained for keratin or CD44, and of cells stained for CD44 alone, for keratin alone, or for both CD44 and keratin.

### Statistical analysis

All data are based on at least three experimental repeats, unless otherwise stated, and are reported as mean ±SEM. P-values were obtained using a Mann-Whitney U test.

## Results

### CD44^high^ESA^low^ EMT CSCs express higher levels of the standard CD44 isoform and lower levels of variant CD44 exons, compared with the CD44^high^ESA^high^ epithelial CSCs

**Figure 1:**
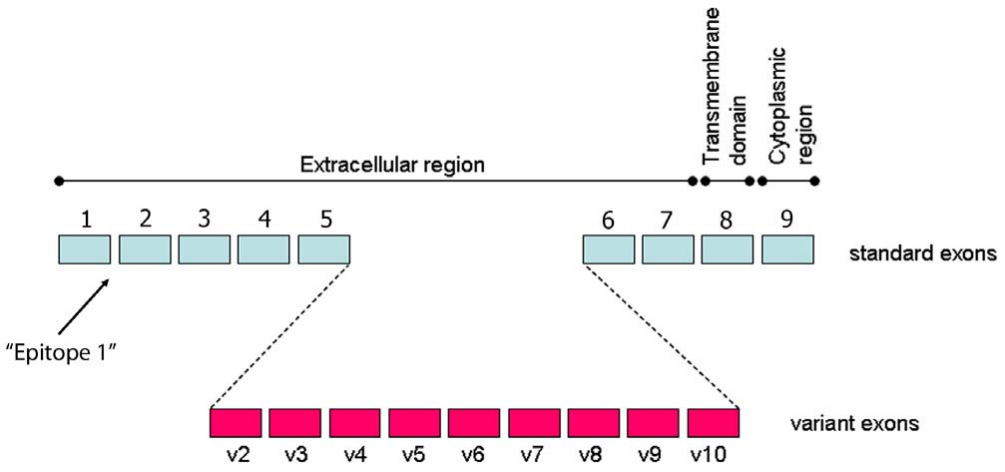
CD44 standard and variant exons. A representation of the 9 standard and 9 variant exons of CD44, showing the names of each variant exon and where they fall in the extracellular region of the protein product. The position of the “Epitope 1” region recognised by the CD44 antibody used in these studies is indicated.

**Figure 2:**
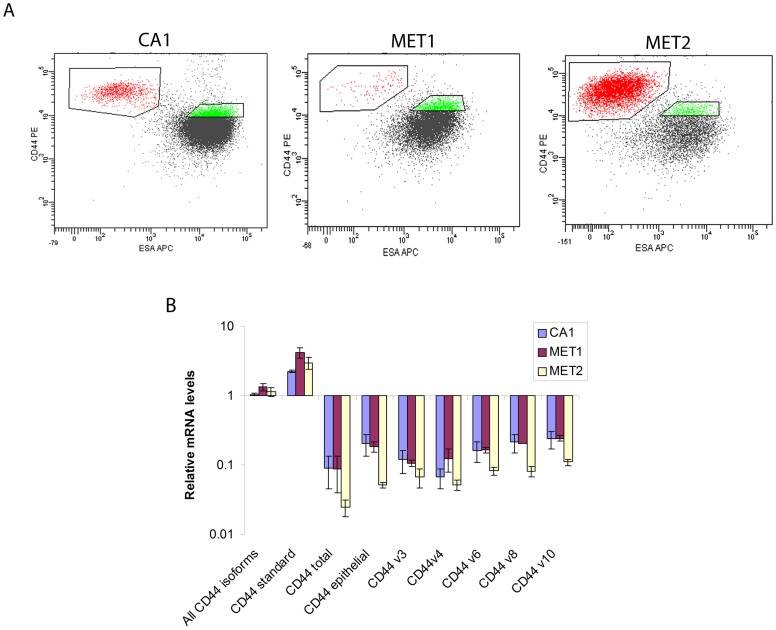
CD44 expression in EMT and non-EMT CSCs. (A) FACS sorting of CA1, Met1 and Met2 cells by expression of CD44 (using the “epitope 1” antibody that detects all forms of CD44) and ESA. The CD44^high^ESA^low^ and CD44^high^ESA^high^ sub-populations are gated and show higher levels of staining of CD44^high^ESA^low^ cells for CD44. (B) QPCR analysis of CD44 variant gene expression in CD44^high^ESA^low^ cells relative to the CD44^high^ESA^high^ cells for the CA1, Met1 and Met2 lines shows marked differences in the isoform expression patterns.

In previous studies, cells with CSC properties have been distinguished from the non-CSC population primarily by their higher levels of CD44 expression. However, the gene coding for the CD44 glycoprotein contains 9 standard and 9 variant exons that generate multiple isoforms (24) (Figure 1). As these influence cell behaviour, it was of interest initially to determine whether differences in isoform expression patterns exist between the EMT (CD44^high^ESA^low^) and non-EMT (CD44^high^ESA^high^) CSC sub-populations that we have previously identified in SCC [32]. The CD44 antibody used in this study and in our previous study [32] binds “epitope 1” present in the distal region of all CD44 isoforms (Figure 1). It therefore recognizes not only the “standard” CD44 isoform, which lacks variant exons, but all other variant CD44 isoforms (CD44v). These include the CD44 “total” isoform, which contains all variant exons, the CD44 “epithelial” isoform, which contains the v8, v9 and v10 variant exons, and other CD44 molecules containing other combinations of variant exons [40], [41]. This antibody was used in combination with an antibody against ESA for an initial flow-cytometric analysis of the CA1, Met1 and Met2 cell lines. Cells brought into suspension using a standard trypsin-EDTA method (PAA) showed higher staining for CD44 of CD44^high^ESA^low^ cell fractions compared to CD44^high^ESA^high^ cells (Figure 2A). It was therefore surprising that QPCR primers that detect all forms of CD44 indicated little difference in the total CD44 mRNA levels between these populations (Figure 2B). However, when CD44 expression was analysed using PCR primers that detect differences in variant isoform expression, it was consistently found that CD44^high^ESA^low^ cells had relatively higher expression of the standard CD44 isoform but lower expression of the CD44 total isoform, the CD44 epithelial isoform, and of individual variant exons v3, v4, v6, v8 and v10.

### Enzyme treatment reduces detection of CD44 variant isoforms

**Figure 3:**
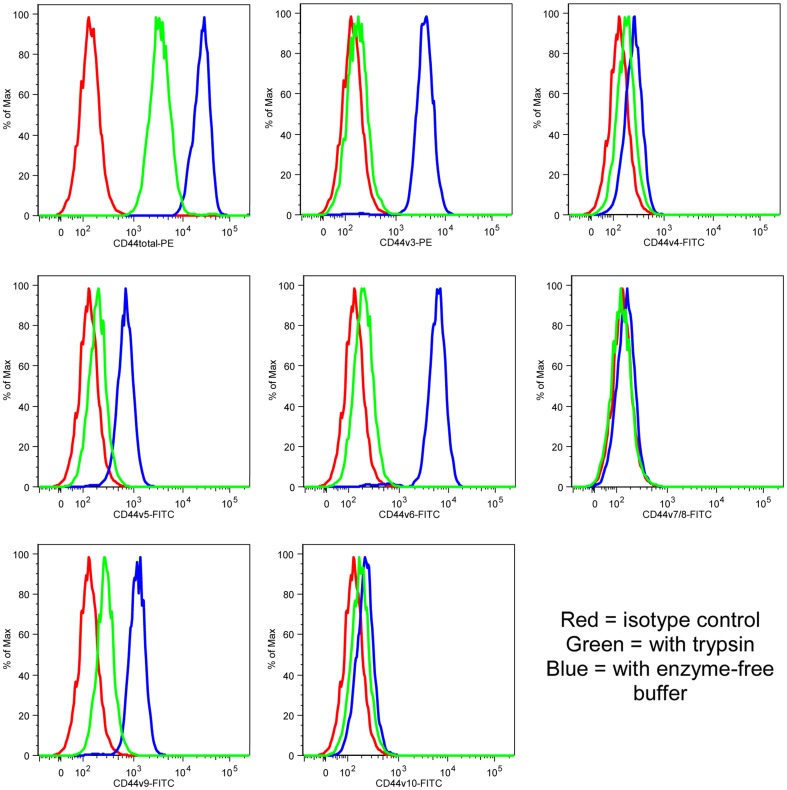
Trypsin treatment prevents cell surface detection of CD44 variant isoforms. Histograms showing expression, assessed by FACS analyses of CA1 cells, of CD44 (“epitope 1” antibody, top left panel) and of CD44 variants after treatment with trypsin (green) or enzyme-free buffer (blue). The isotype control is in red.

As trypsin treatment has been reported to destroy some CD44 cell surface epitopes [36], we examined what effects various cell dissociation methods used to bring cells into suspension have on CD44 staining patterns subsequently detected by flow cytometry. CA1 cells were released from adherent culture using trypsin or enzyme-free buffer and then stained with the “epitope 1” antibody or antibodies against several individual CD44 variants (Figure 3 and Appendix S3). Epitopes encoded by v4, v7/8 and v10 were never detected but those encoded by v3, v5, v6 and v9 were strongly detected on cells released with enzyme-free buffer whereas only very little signal was detected on cells released with trypsin, indicating sensitivity of the variant isoforms to trypsin.

**Figure 4:**
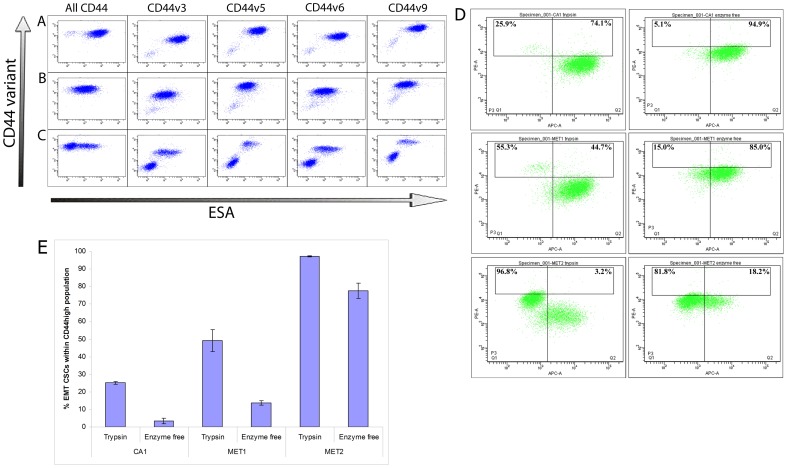
Detection of CD44 variant isoform expression on the CD44^high^ESA^high^ cell sub-population. FACS analysis of CD44 variant isoform expression on cells of the CA1 (A), Met1 (B) and Met2 (C) cell lines isolated using enzyme-free buffer showing the CD44 variants on the y-axis and ESA expression on the x-axis. For all cell lines, similar total levels of CD44 are detected on the CD44^high^ESA^low^ and CD44^high^ESA^high^ cells but lower levels of the v3, v5, v6 and v9 isoforms are detected on the CD44^high^ESA^low^ cells. (D) FACS analysis of CD44 (y-axis) and ESA (x-axis) expression on CA1 (top), Met1 (middle) and Met2 (bottom) cells treated with either trypsin (left) or enzyme-free buffer (right). Loss of detection of CD44 variant isoforms after trypsin treatment results in a large increase in the fraction of motile CSCs within the 5% of cells showing the highest CD44 expression. Representative plots. (E) Quantification of the experiments depicted in figure 4D, showing the percentage of EMT CSCs within the 5% CD44^high^ population after treatment with trypsin or enzyme-free buffer.

CA1, Met1 and Met2 cells released with enzyme-free buffer and co-stained for CD44 variants and ESA (Figure 4 A, B and C) confirmed that the CD44^high^ESA^low^ cells express lower levels of the v3, v5, v6 and v9 variant proteins. When variant CD44 isoforms were conserved, CD44^high^ESA^low^ cells were not in fact much higher than the bulk population of cells for CD44 staining (Figure 4D). Further, it can be seen that if the top 5% of cells are selected based on CD44 staining alone, the composition of this CD44^high^ population changes markedly depending on which isolation method is used, with a much higher proportion of the population being comprised of EMT CSCs if enzymatic isolation is used. Thus it is the stability of the standard CD44 isoform and its increased expression on the CD44^high^ESA^low^ EMT CSCs that results in enrichment of these cells within the CD44^high^ population after enzymatic isolation with trypsin.

**Figure 5:**
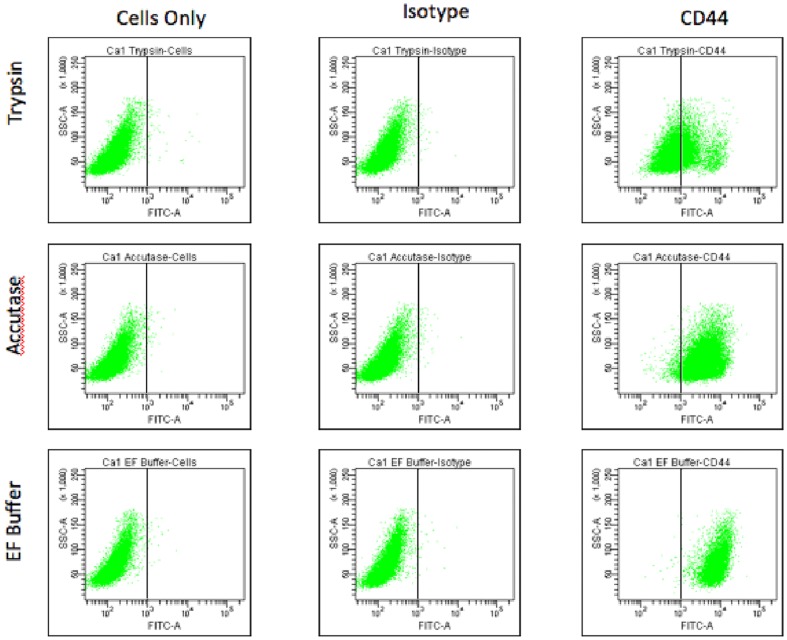
Enzyme treatment decreases the size of the CD44-positive population. Following isolation with trypsin, Accutase or enzyme-free (EF) buffer, CA1 cells were subject to FACS analysis either unstained, stained with an isotype control, or stained for CD44. Unstained and isotype-stained cells were unaffected by method of isolation but marked differences in the proportion of cells classified as staining positive for CD44 are seen.

To visualise the effect of different cell dissociation methods on the percentage of cells reported as CD44-positive in flow cytometry, we dissociated the CA1 cell line from culture using either trypsin, enzyme-free buffer, or Accutase (a proprietary enzymatic solution claimed by the manufacturer to have less general proteolytic activity than trypsin) and then stained for CD44 (Figure 5 and Appendix S3). The different isolation methods had no effect on isotype control staining, but there was a marked difference in the proportion of cells staining CD44-positive using the different methods. The enzymatic destruction of CD44 variants therefore has a profound effect on the percentage of cells that are reported as CD44-positive in analysis of cells after enzymatic dissociation.

### Enzymatic dissociation reduces detection of CD44-positive cells in fresh tumour specimens

**Figure 6:**
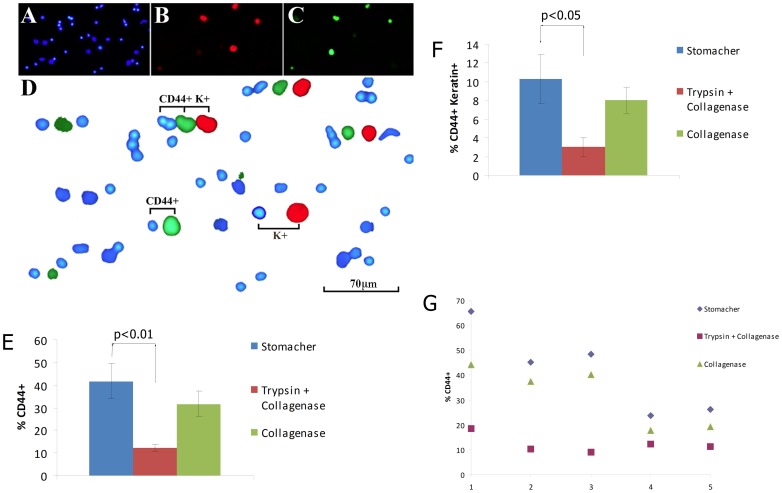
Differential staining for CD44 on cells isolated from fresh tumour samples using different methods. Panels A–D illustrate the procedure used to identify staining patterns. Cell smears were prepared and co-stained with DAPI (A) with a pan-keratin antibody (B) and with the CD44 “epitope 1” antibody (C). These panels show images manipulated by adjusting capture thresholds in PhotoShop to identify cells with set levels of staining above a background level. For each field, this level was held constant to generate the three images, one for each fluorochrome, containing only the stained cells. These images were then combined, slightly out of lateral register, to allow identification of cells with absent or low levels staining for CD44 or keratin (blue nuclei only), staining for CD44 only (CD44+), keratin only (K+), or both CD44 and keratin (CD44+K+). Treatment with trypsin+collagenase or collagenase alone consistently reduced the fraction of cells that was positive for CD44 (E) and for both CD44 and keratin (F). Displaying the 5 assayed tumours individually (G) demonstrates the striking variation in CD44 staining between tumours, and that this variation is reduced following enzymatic treatment. Statistical analysis was conducted using a Mann-Whitney test.

The staining of CD44 expressing cells in cell populations isolated from fresh biopsies of head and neck SCC also varied with the method of isolation. As shown in Figure 6, cell smears were co-stained with DAPI, a pan-keratin antibody, and the CD44 “epitope 1” antibody. This enabled counts of cell nuclei and classification of cells as stained or unstained for CD44. The keratin antibody was used to mark epithelial cells. We compared two commonly used enzymatic isolation methods, trypsin plus collagenase [10], [16] and collagenase alone [3], [12], [15], with extraction without enzymes. Cell counts indicated variation in the size of both the fraction of the total cells classified as positive for CD44 and in the size of the cell fraction positive for both CD44 and keratin (Fig. 6 E,F). Staining for CD44 indicated that the stained fraction of cells was significantly higher after extraction without enzymes than after extraction with trypsin plus collagenase (CD44 positive: Mann–Whitney *U* = 0, *n*_1_ = *n*_2_ = 5, *P*<0.01 two-tailed; CD44 positive+Keratin positive: Mann–Whitney *U* = 2, *n*_1_ = *n*_2_ = 5, *P*<0.05 two-tailed). Staining was also somewhat reduced by extraction with collagenase alone, although this was not statistically significant. As has been noted previously [12], we also witnessed a large variation in the percentage of CD44-positive cells between tumours (Fig. 6G). However, treatment with trypsin plus collagenase or collagenase alone consistently caused a decrease in the percentage of cells stained CD44-positive across all tumours and also caused a decrease in the variation in CD44 staining between tumours.

## Discussion

Previous reports concerning isolation of CSCs from tumours and tumour cell lines have described the use of a range of stem-cell-related markers but expression of CD44, alone or together with additional markers, has been widely used for the identification of CSCs in breast, oral, prostate, colon and pancreatic malignancies [2], [3], [17]–[19]. The CD44 antibody employed in the present and most previous studies of CSCs [2], [3], [18], [32], [35] recognizes all CD44 isoforms and consequently does not indicate how total levels are influenced by variation in patterns of isoform expression. In the present study we found, using flow cytometry, that the total levels of CD44 expression detected for cells isolated by non-enzymatic methods were similar for EMT and non-EMT cells. However, the pattern of isoform expression differed with the standard CD44 isoform being more highly expressed by the EMT CSCs and variant CD44 isoforms more highly expressed by the non-EMT CSCs. Cell dissociation using trypsin reduced the CD44 staining of the non-EMT CSCs, with a consequent enrichment for EMT CSCs in the CD44^high/+^ population. This is due to the greater proteolytic sensitivity of the variant CD44 isoforms, which are destroyed by trypsin treatment whilst the standard CD44 isoform is preserved. The same effect was demonstrated during cell isolation from fresh tumour specimens, in which commonly used methods of enzymatic cell dissociation caused a reduction in cell staining for CD44 when compared with a non-enzymatic dissociation method.

The reduction in CD44 staining resulting from treatment with proteolytic enzymes caused a reduction both in the representation of epithelial CSCs within the CD44^high^ population and the percentage of the total epithelial cancer cell population staining CD44-positive. Previously, some studies using CD44 as a CSC marker have concentrated only on the CD44^high^ population [2], [32], [35] whereas others have isolated the entire CD44-positive population from tumours [3], [12]. Our results show that the method of cell dissociation will effect the composition of the isolated cell population in both types of study. The observed reduction, and reduced variation between tumours, of CD44-positive staining after enzymatic dissociation may be of particular significance for studies that aim to use CD44-positive staining as a prognostic indicator [12].

Analyses of the properties of CSCs are largely dependent on the ability to accurately identify and assay them. We have previously demonstrated the existence of two different CD44^high^ CSCs in SCC, one a fast-growing non-EMT CSC with an epithelial phenotype and the other an invasive EMT CSC with a mesenchymal phenotype [32]. The demonstration that the two different CSC sub-populations express CD44 isoforms that are differentially affected by isolation procedures, resulting in varying levels of enrichment of the two CSC types within the CD44^high^ fraction, has implications for studies involving isolation or characterization of CSCs based on their expression of CD44. EMT and non-EMT CSCs exhibit very different behaviour both *in vitro* and *in vivo* [32]. It is therefore critical that consideration be given to use of multiple CSC markers and suitable procedures for cell isolation in order that the correct populations are assayed in studies of CSCs.

## Supporting Information

Appendix S1 (DOC)

Appendix S2 (TIF)

Appendix S3 (DOC)
