# Supplementary material for: CD44 Staining of Cancer Stem-Like Cells Is Influenced by Down-Regulation of CD44 Variant Isoforms and Up-Regulation of the Standard CD44 Isoform in the Population of Cells That Have Undergone Epithelial-to-Mesenchymal Transition
Source: PLoS One. 2013 Feb 20;8(2):e57314. doi: 10.1371/journal.pone.0057314 (PMC3577706; doi:10.1371/journal.pone.0057314)
Supplement: Appendix S1 — (DOC) [file pone.0057314.s001.doc]

**Appendix S1.**

Primer sequences and binding sites:

**All CD44**

F: CGGACACCATGGACAAGTTT, binds within standard exon 1.

R: GAAAGCCTTGCAGAGGTCAG, binds within standard exon 2.

**CD44 standard**

F: AGCAGCGGCTCCTCCAGTGA, binds within standard exon 5.

R: CCCACTGGGGTGGAATGTGTCT, binds within standard exon 6.

These primers give a 162bp product from the standard CD44 isoform. The smallest possible product that can be obtained with incorporation of variant exons is 252bp (with incorporation of 90bp exon v9), which is above the maximum product size that can be efficiently amplified (Power SYBR Green User Guide, Applied Biosystems). The dissociation curve for these primers (below) indicates only one product, demonstrating that only the standard isoform is detected.


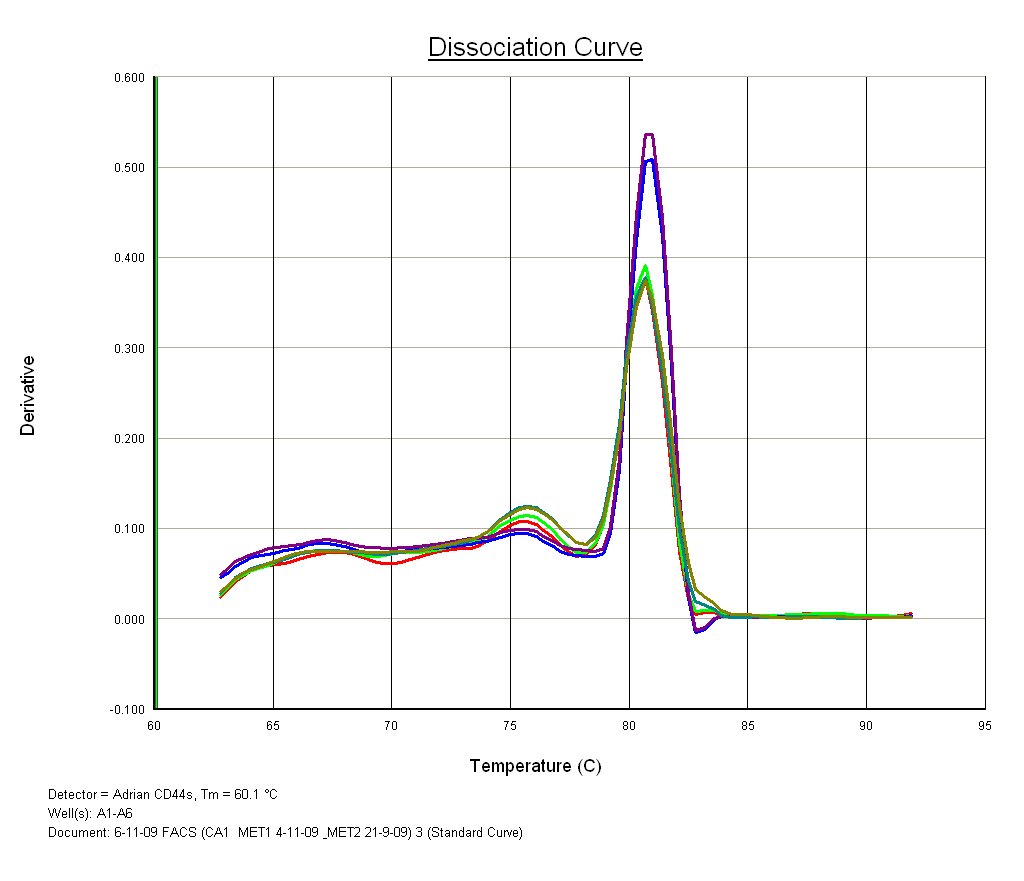


**CD44 total**

F: AGCAACCAAGAGGCAAGAAA, binds within variant exon v2.

R: GTGTGGTTGAAATGGTGCTG, binds across variant exons v3 and v4 (spanning the exon-exon junction).

The combination of exons v2, v3 and v4 together is only found in the CD44 total isoform [1]. This is confirmed by the dissociation curve (below), which shows only one product.


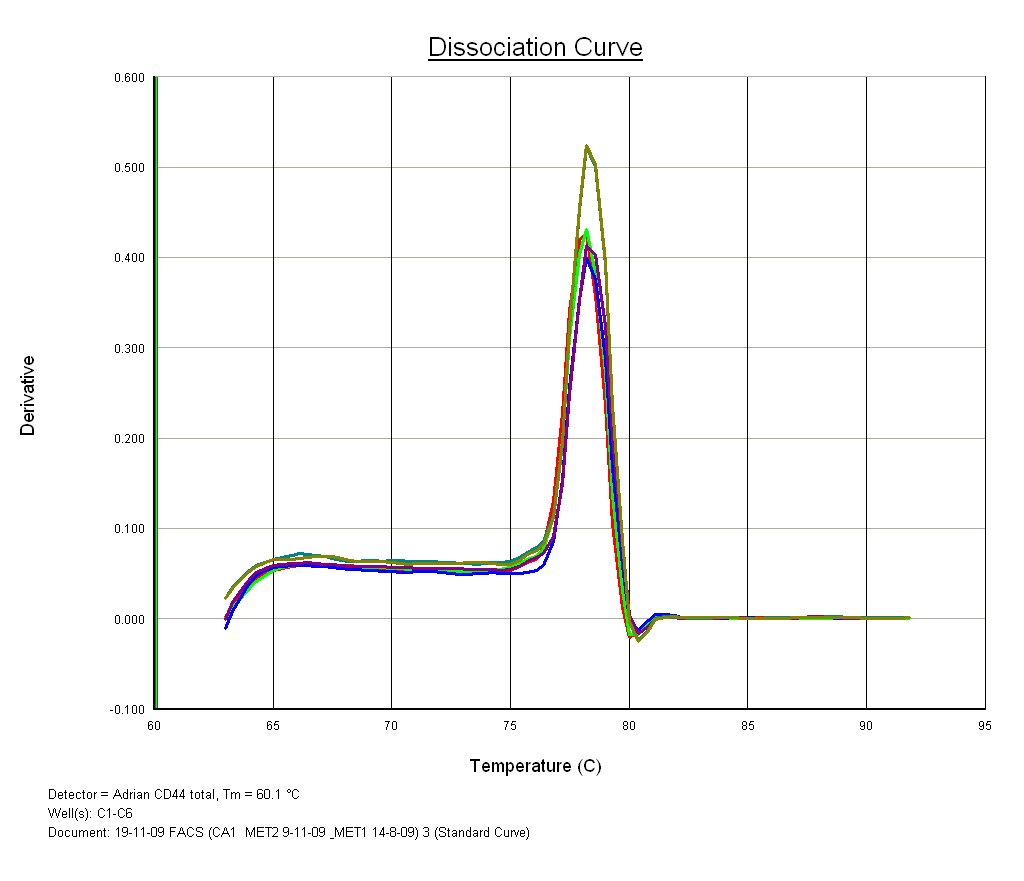


**CD44 epithelial**

F: GAAAGGAGCAGCACTTCAGG, binds within standard exon 5.

R: GAGGTCCTGTCCTGTCCAAA, binds within variant exon v8.

The transition from standard exon 5 to variant exon v8, with all exons between omitted, is a feature of the CD44 epithelial isoform [2]. These primers give a 202bp product from the epithelial CD44 isoform. The smallest possible product that can be obtained with incorporation of other variant exons in between is 314bp (with incorporation of 114bp exon v4), which is above the maximum product size that can be efficiently amplified (Power SYBR Green User Guide, Applied Biosystems). The dissociation curve for these primers (below) indicates only one product, demonstrating that only the epithelial isoform is detected.


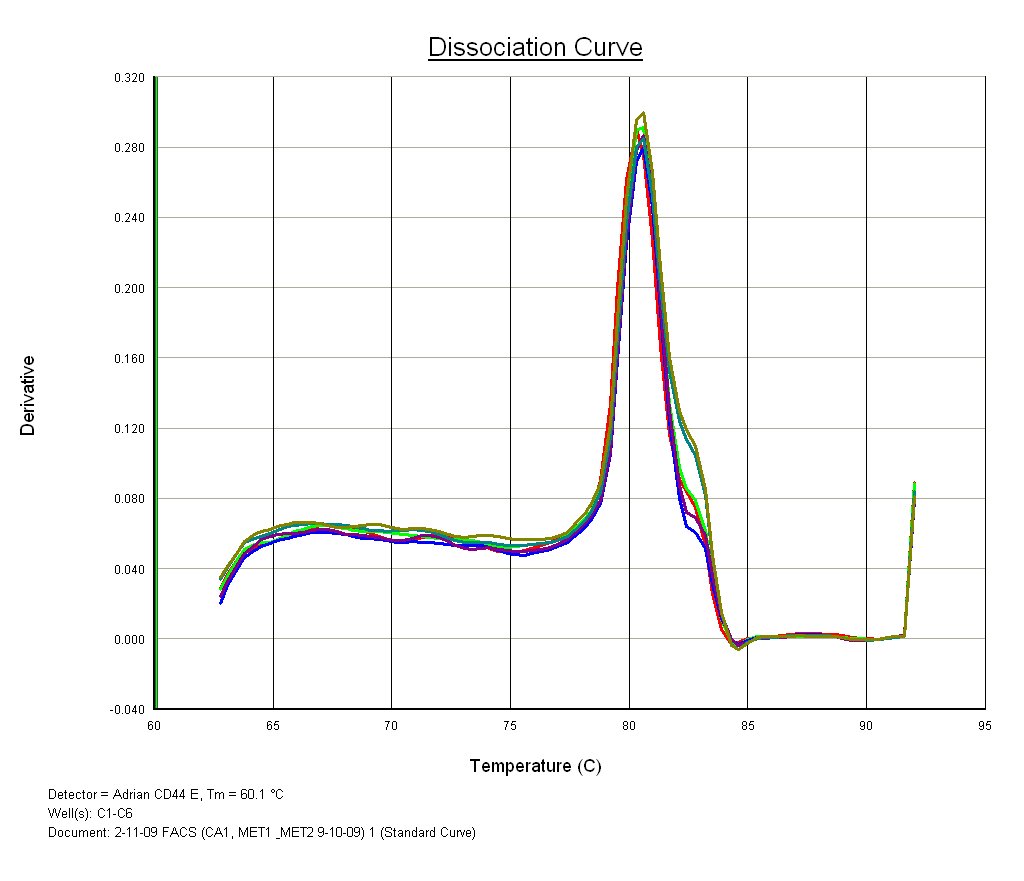


**CD44v3**

F: GCACTTCAGGAGGTTACATC, binds within standard exon 5.

R: CTGAGGTGTCTGTCTCTTTC, binds within variant exon v3.

**CD44v4**

F: ACCACACCACGGGCTTTTGACC, binds within variant exon v4.

R: GGGTTCCACTGGGTCCAGTCCT, binds within variant exon v4.

**CD44v6**

F: AGGAACAGTGGTTTGGCAAC, binds within variant exon v6.

R: CGAATGGGAGTCTTCTCTGG, binds within variant exon v6.

**CD44v8**

F: TCAGCCTACTGCAAATCCAA, binds within variant exon v8.

R: GAGGTCCTGTCCTGTCCAAA, binds within variant exon v8.

**CD44v10**

F: GGAATGATGTCACAGGTGGA, binds within variant exon v10.

R: AGGTCACTGGGATGAAGGTC, binds within variant exon v10.

**GAPDH**

F: GTGAACCATGAGAAGTATGACAAC

R: CATGAGTCCTTCCACGATACC

1. Friedrichs K, Franke F, Lisboa BW, Kugler G, Gille I, et al. (1995) CD44 isoforms correlate with cellular differentiation but not with prognosis in human breast cancer. Cancer Res 55: 5424-5433.

2. Herold-Mende C, Seiter S, Born AI, Patzelt E, Schupp M, et al. (1996) Expression of CD44 splice variants in squamous epithelia and squamous cell carcinomas of the head and neck. J Pathol 179: 66-73.
