# Supplementary figures and images for: CD44 Staining of Cancer Stem-Like Cells Is Influenced by Down-Regulation of CD44 Variant Isoforms and Up-Regulation of the Standard CD44 Isoform in the Population of Cells That Have Undergone Epithelial-to-Mesenchymal Transition
Source: PLoS One. 2013 Feb 20;8(2):e57314. doi: 10.1371/journal.pone.0057314 (PMC3577706; doi:10.1371/journal.pone.0057314)

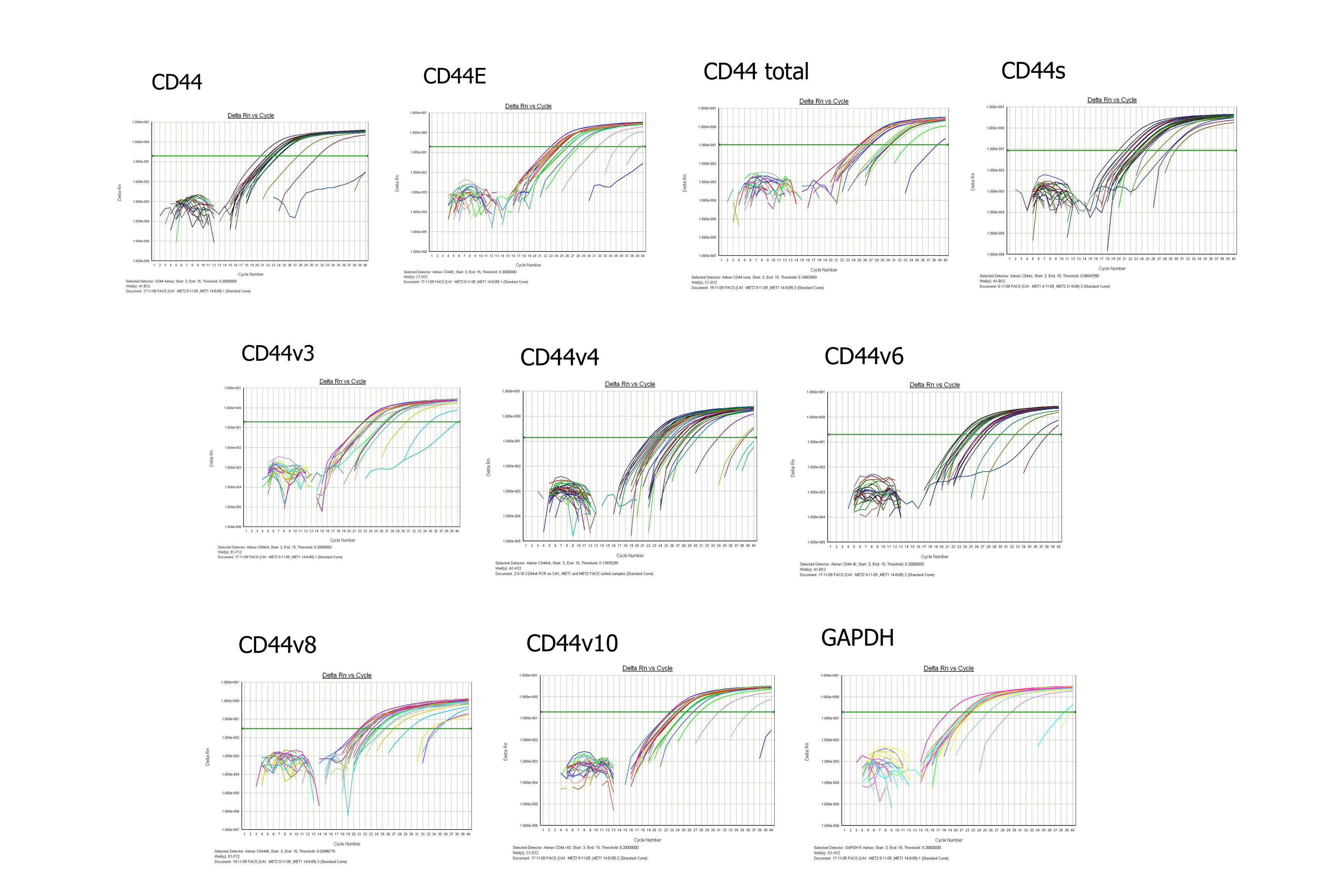

Supplement: Appendix S2 — (TIF) [file pone.0057314.s002.tif]
