# Supplementary material for: CD44 Staining of Cancer Stem-Like Cells Is Influenced by Down-Regulation of CD44 Variant Isoforms and Up-Regulation of the Standard CD44 Isoform in the Population of Cells That Have Undergone Epithelial-to-Mesenchymal Transition
Source: PLoS One. 2013 Feb 20;8(2):e57314. doi: 10.1371/journal.pone.0057314 (PMC3577706; doi:10.1371/journal.pone.0057314)
Supplement: Appendix S3 — (DOC) [file pone.0057314.s003.doc]

**Appendix S3**

**A) Tabulated data for all repeat experiments relating to Figure 3**

|  | **Test fluorescent units minus isotype fluorescent units** | | |  |  |
| --- | --- | --- | --- | --- | --- |
|  | **Repeat 1** | **Repeat 2** | **Repeat 3** | **Average** | **SEM** |
| **Trypsin all CD44** | 7233 | 6986 | 4024 | 6081 | 1030.968638 |
| **Trypsin v3** | 71 | 56 | 67 | 64.66666667 | 4.484541349 |
| **Trypsin v4** | 58 | 123 | 272 | 151 | 63.34298172 |
| **Trypsin v5** | 294 | 292 | 73 | 219.6666667 | 73.33560603 |
| **Trypsin v6** | 38 | 38 | 82 | 52.66666667 | 14.66666667 |
| **Trypsin v7/8** | 15 | 4 | 0 | 6.333333333 | 4.484541349 |
| **Trypsin v9** | 197 | 17657 | 8710 | 8854.666667 | 5040.786854 |
| **Trypsin v10** | 12 | 21 | 52 | 28.33333333 | 12.1151879 |
| **Enzyme free all CD44** | 42392 | 41192 | 27003 | 36862.33333 | 4941.822887 |
| **Enzyme free v3** | 1535 | 1557 | 4051 | 2381 | 835.0241513 |
| **Enzyme free v4** | 175 | -1345 | 619 | -183.6666667 | 594.6442447 |
| **Enzyme free v5** | 20855 | 22100 | 650 | 14535 | 6951.79653 |
| **Enzyme free v6** | 5673 | 5956 | 5981 | 5870 | 98.76402854 |
| **Enzyme free v7/8** | 7 | 24 | 30 | 20.33333333 | 6.887992773 |
| **Enzyme free v9** | 1175 | 59806 | 76657 | 45879.33333 | 22875.36604 |
| **Enzyme free v10** | -21 | 51 | 141 | 57 | 46.86149806 |

|  | **Ratio of enzyme free buffer:trypsin signal** | | |  |  |
| --- | --- | --- | --- | --- | --- |
|  | **Repeat 1** | **Repeat 2** | **Repeat 3** | **Average** | **SEM** |
| **all CD44** | 5.86091525 | 5.896364157 | 6.710487 | 6.155922 | 0.277471 |
| **v3** | 21.61971831 | 27.80357143 | 60.46269 | 36.62866 | 12.04997 |
| **v4** | 3.017241379 | -10.93495935 | 2.275735 | -1.88066 | 4.532207 |
| **v5** | 70.93537415 | 75.68493151 | 8.90411 | 51.84147 | 21.51242 |
| **v6** | 149.2894737 | 156.7368421 | 72.93902 | 126.3218 | 26.77782 |
| **v7/8** | 0.466666667 | 6 | 20.3 | 8.922222 | 5.908886 |
| **v9** | 5.964467005 | 3.387098601 | 8.801033 | 6.050866 | 1.563465 |
| **v10** | -1.75 | 2.428571429 | 2.711538 | 1.130037 | 1.442333 |

The numbers in the bottom table were calculated from those in the top table; for each repeat experiment the value obtained using enzyme free buffer was divided by the value obtained using trypsin to give a ratio or fold difference.

**B) Tabulated data for all repeat experiments relating to Figure 5**

|  | **Test fluorescent units minus isotype fluorescent units** | | |  |  |
| --- | --- | --- | --- | --- | --- |
|  | **Repeat 1** | **Repeat 2** | **Repeat 3** | **Average** | **SEM** |
| **Trypsin CD44** | 1045 | 968 | 1008 | 1007 | 22.23360819 |
| **Accutase CD44** | 2830 | 2883 | 4115 | 3276 | 419.7789101 |
| **Enzyme free CD44** | 8212 | 7655 | 8226 | 8031 | 188.0434347 |
